# Nachsorge nach Steinsanierung bei Urolithiasis

**DOI:** 10.1007/s00120-022-01816-5

**Published:** 2022-04-05

**Authors:** Kevin Stritt, Piet Bosshard, Beat Roth

**Affiliations:** 1 Department of Urology, Lausanne University Hospital (CHUV), University of Lausanne, Lausanne, Schweiz; 2 Service d’urologie, CHUV | Centre hospitalier universitaire vaudois, Rue de Bugnon 46, 1011 Lausanne, Schweiz

**Keywords:** Harnsteine, Bildgebung, Steinmetaphylaxe, Rezidiv, SWL, Kidney stone, Imaging, Metaphylaxis, Recurrence, SWL

## Abstract

**Hintergrund:**

Von einer Harnsteinerkrankung sind häufig auch jüngere Menschen betroffen. Weil das Rezidivrisiko hoch ist, ist es wichtig, gefährdete Personen regelmäßig nachzukontrollieren.

**Ziel:**

Es wird das Ausmaß von Harnsteinleiden und dessen gesundheitlicher und wirtschaftlicher Einfluss in der Bevölkerung untersucht und eine Empfehlung zur allgemeinen und steinspezifischen Nachkontrolle gegeben.

**Material und Methode:**

Publikationen sowie Leitlinienempfehlungen werden analysiert und diskutiert.

**Ergebnisse:**

Das Rezidivrisiko nach einem Steinerstereignis kann je nach Risikoprofil hoch sein. Eine erste metabolische Abklärung ist rasch nach einer Steintherapie durchzuführen. Eine allgemeine Ernährungsanpassung sollte durch eine steinspezifische je nach Risikoprofil intensiviert werden. Eine Ernährungsberatung kann dabei hilfreich sein. Bildgebende Kontrollen nach Steintherapie erfüllen den Zweck der Kontrolle des Therapieerfolgs sowie der Früherkennung von Rezidiven. Da das Rezidivrisiko je nach Steinzusammensetzung stark variieren kann, sollte nicht nur die Art der Bildgebung, sondern auch deren Frequenz dementsprechend angepasst werden. Dasselbe gilt auch für die verschiedenen Steintherapien, welche die Häufigkeit und die Art der bildgebenden Nachkontrollen mitbestimmen. Genaue Richtlinien sowie Kosteneffizienzanalysen von Nachkontrollen nach Steintherapie fehlen leider.

**Schlussfolgerung:**

Die akute Urolithiasis stellt ein einschneidendes Erlebnis für die Patienten dar. Dementsprechend ist deren Bereitschaft zur Metaphylaxe und Nachkontrolle kurz nach dem Ereignis groß. Da das Rezidivrisiko nach Steinerstereignis sehr hoch sein kann, ist eine gute Nachbetreuung nach Steintherapie mit regelmäßigen Nachkontrollen unerlässlich. Die Frequenz der Nachkontrollen sollte der Wahrscheinlichkeit für ein Steinrezidiv angepasst werden.

## Abstract (en)

**Background:**

Urinary stones often affect younger people. Because the risk of recurrence is high, regular follow-up is important for individuals at risk.

**Objective:**

To summarize the extent of urinary stones and the health and economic impact in the population; to provide recommendations for general and stone-specific follow-up.

**Materials and methods:**

Analysis and discussion of publications and guideline recommendations.

**Results:**

The risk of recurrence after a stone attack can be high depending on the risk profile. An initial metabolic workup should be performed promptly after stone therapy. General dietary management should be intensified by stone-specific dietary management depending on the risk profile. Nutritional counseling may be helpful. Imaging after stone therapy is used to monitor the success of treatment and detect recurrences early. Since the risk of recurrence can vary greatly depending on the stone composition, not only the type of imaging but also its frequency should be adjusted accordingly. The same applies to the various stone therapies, which help determine the frequency and type of imaging follow-up. Exact guidelines and cost-effectiveness analyses of follow-up examinations after stone therapy are unfortunately missing.

**Conclusions:**

Acute urolithiasis represents an excruciating experience for patients. Accordingly, their willingness to undergo metaphylaxis and follow-up shortly after the event is strong. Since the risk of recurrence after a stone attack can be very high, regular follow-up after stone therapy is essential. The frequency of follow-up should be adapted to the probability of stone recurrence.

Die Inzidenz und Prävalenz des Harnsteinleidens (Urolithiasis) nimmt weltweit zu, unabhängig von Alter, Geschlecht und ethnischer Zugehörigkeit [1]. Demzufolge ist die Therapie von Harnsteinpatienten aus dem täglichen Alltag des Urologen nicht mehr wegzudenken. Dies umso mehr, als bei der Urolithiasis häufig gilt: „Nach der Therapie ist vor der Therapie“, liegen doch die Rezidivraten nach initialem Steinereignis ohne spezifische Behandlung bei 10–20 % innerhalb eines Jahres, 40 % innerhalb von 5 und 75 % innerhalb von 20 Jahren [2]. Nach Rezidivsteinereignissen liegen diese Zahlen noch deutlich höher. Umso wichtiger sind die richtige Beratung zur Steinmetaphylaxe sowie die regelmäßige Nachsorge, welche in diesem Artikel beschrieben wird.

Die Inzidenz und Prävalenz des Harnsteinleidens (Urolithiasis) nimmt weltweit zu

Die Urolithiasis stellt ein zunehmendes Gesundheitsproblem dar, besteht doch in der westlichen Welt eine Lebenszeitinzidenz von bis zu 18,8 % bei Männern und 9,4 % bei Frauen [1]. Dementsprechend kann man von einer Zivilisationskrankheit sprechen, die auch mit der eiweißreichen Kost zu tun hat und demzufolge in ärmeren Ländern bedeutend seltener ist. In den USA betragen die Kosten von Krankenhausaufenthalten, Operationen und Arbeitsabwesenheiten im Zusammenhang mit Urolithiasis mehr als 5 Mrd. $/Jahr [3].

Die Urolithiasis wird oft bagatellisiert, obwohl sie eine eigenständige Diagnose darstellt und die zugrunde liegende Ursache gesucht werden sollte. Wichtige Erkrankungen wie ein metabolisches Syndrom, Diabetes mellitus Typ 2, Bluthochdruck und Osteoporose können damit verbunden sein. Viele Faktoren begünstigen die Bildung von Harnsteinen; die wichtigsten Faktoren sind genetische Varianten und Umweltbedingungen, insbesondere Ernährungsfaktoren. Die Urolithiasis kann daher als eine systemische Erkrankung betrachtet werden, die eine spezifische Behandlung erfordert. Aus diesem Grund sollten Patienten mit rezidivierenden Nierensteinerkrankungen oder signifikanten Begleiterkrankungen zu einer spezialisierten Konsultation überwiesen werden. Die Arbeitsgruppe für das nationale, schweizweite Nierensteinregister (Swiss Kidney Stone Cohort [SKSC]; www.nccr-kidney.ch) hat dementsprechende Empfehlungen abgegeben (Tab. 1). Die Risikofaktoren, welche einer weiterführenden metabolischen Abklärung bedürfen, entsprechen dabei weitestgehend denen der Hochrisikogruppe, wie sie in den Europäischen Richtlinien beschrieben werden. Auf diese Weise kann eine metabolische Abklärung durchgeführt und eine personalisierte Behandlung vorgeschlagen werden, um das Risiko eines erneuten Auftretens von Harnsteinen zu reduzieren und die frühzeitige Diagnose und Behandlung von Begleiterkrankungen zu unterstützen.

**Table 1:** 

*1. Wiederkehrende Nierensteinepisoden (>* *1)*
*2. Einzelne Nierensteinepisode mit mindestens einem der folgenden Risikofaktoren:*
Erste Präsentation im Alter von < 35 Jahren
Positive Familiengeschichte
Nicht-Kalziumoxalatsteine
Gastrointestinale Erkrankungen (z. B. Magenbypassoperationen, chronisch entzündliche Darmerkrankungen, Malabsorption)
Osteoporose
Nephrokalzinose oder Markschwammniere
Einzelniere
Auftreten in der Schwangerschaft
Gicht
Metabolisches Syndrom
Restkonkrement (> 3 Monate nach Therapie)
Bilaterale oder mehrere Steine
Chronischer Harnwegsinfekt
Chronische Niereninsuffizienz
Nierentransplantation

## Metabolische Nachsorge und Metaphylaxe

Grundlage der metabolischen Abklärung ist die chemische Harnsteinzusammensetzung. Aus diesem Grund sollten spontan ausgeschiedene oder im Rahmen von Interventionen asservierte Konkremente einer Harnsteinanalyse zugeführt werden. Eine Harnsteinanalyse ist auch bei Rezidivsteinen empfohlen, da sich die Harnsteinzusammensetzung im Verlauf ändern kann [4]. Eine Basisdiagnostik wird für alle Harnsteinpatienten empfohlen. Sie ermöglicht die Zuordnung des Steinpatienten zur Niedrig- oder Hochrisikogruppe. Neben der Harnsteinanalyse gehören die folgenden Untersuchungen zur obligaten Basisdiagnostik: Anamnese, klinische Untersuchung (inklusive Sonographie der Niere), Blutlabor (Elektrolyte, Harnsäure, Harnstoff, Kreatinin) und Urinuntersuchung (Sediment, Urinkultur).

Eine Basisdiagnostik ermöglicht die Zuordnung des Steinpatienten zur Niedrig- oder Hochrisikogruppe

Die erweiterte metabolische Diagnostik wird bei Patienten mit hohem Rezidivrisiko durchgeführt (Tab. 1). Sie stützt sich neben einer Blutuntersuchung auf die Analyse von zwei 24-h-Sammelurinen, um die Ausscheidung von lithogenen und inhibitorischen Substanzen im Urin zu analysieren. Um unverfälschte Ergebnisse zu erhalten, sollte der Patient zum Zeitpunkt der Untersuchung möglichst steinfrei sein und die letzte Intervention mindestens 3 Wochen zurückliegen [5]. Bei Patienten, die eine medikamentöse Metaphylaxe erhalten, sollte innerhalb von 3–6 Monaten eine Nachuntersuchung inklusive Auswertung eines 24-h-Sammelurins durchgeführt werden, um den Therapieerfolg zu verifizieren. Bei Therapieerfolg sind weitere metabolische Kontrolluntersuchungen alle 12 Monate ausreichend [6, 7].

Die generellen Empfehlungen zur allgemeinen Harnsteinmetaphylaxe gelten grundsätzlich für alle Harnsteinpatienten (Tab. 2; [6]). Die Erhöhung der täglichen Flüssigkeitszufuhr ist die wichtigste Metaphylaxe. Sie zielt darauf ab, die Konzentration lithogener Salze im Urin und deren Übersättigung durch eine regelmäßige Verdünnung des Urins über 24 h zu verringern. Eine randomisierte kontrollierte Studie zeigte, dass ein Urinvolumen von > 2 l/Tag zu einer erheblichen Verringerung des Rezidivrisikos nach 5 Jahren führt [8]. Die Flüssigkeitszufuhr sollte hierbei gleichmäßig über 24 h verteilt werden, damit Konzentrationsspitzen der lithogenen Substanzen vermieden werden können [9].

Diese einfache und kostengünstige Maßnahme hat sich als sehr wirksam erwiesen und sollte daher allen Patienten vermittelt werden. Die Ernährungsberatung sollte sich im Wesentlichen auf 4 Punkte konzentrieren: 1) Eine ausreichende Kalziumzufuhr über die Ernährung (1000 mg/Tag, hauptsächlich in Form von Milchprodukten; [10]); 2) eine Beschränkung der übermäßigen Kochsalzzufuhr (maximal 5 g NaCl/Tag, entsprechend den Empfehlungen der Weltgesundheitsorganisation); 3) eine Beschränkung der Aufnahme von tierischem Eiweiß (übermäßige Aufnahme ab 1 g/kgKG und Tag; Ziel: ≤ 0,8 g/kg und Tag; [11]) und 4) eine Reduzierung der Oxalataufnahme mit der Nahrung, insbesondere der zwanghaften Aufnahme (übermäßige Aufnahme über einen kurzen Zeitraum).

**Table 2:** 

*Harnvolumen↑*
≥ 2 l/Tag
Getränke gleichmäßig über 24 h verteilt (vor dem Schlafengehen trinken!)
*Kalzium↑*
Keine Einschränkung
1000 mg/Tag Kalzium (100 g Hartkäse = 1000 mg Kalzium; 100 ml Milch = 120 mg Kalzium)
Kalziumzufuhr bevorzugt während der Mahlzeiten, um eine Hyperoxalurie zu vermeiden (Bindung des Nahrungsoxalats im Darmlumen, wodurch seine Absorption verhindert wird)
*Salz↓*
Etwa 5 g/Tag
*Tierisches Eiweiß↓*
5 ×/Woche, nie 2‑mal am selben Tag
Maximale tägliche Aufnahme ≤ 1 g/kgKG
*Oxalat↓*
Vermeiden von übermäßigem Konsum von oxalatreichen Getränken (schwarzer/grüner Tee, Eistee) oder Nahrungsmitteln (Spinat, Erdnüsse, Walnüsse, Rhabarber, Rote Beete usw.)

Die steinartspezifische Abklärung und Rezidivprophylaxe ergänzt die allgemeine Harnsteinmetaphylaxe, welche nur für Patienten der Hochrisikogruppe erforderlich ist [9]:

### Kalziumoxalat- und Kalziumphosphatsteine

Die metabolische Diagnostik bei Kalziumphosphatsteinbildnern ist identisch mit der von Kalziumoxalatsteine (Parathormon zur Bestätigung oder zum Ausschluss eines Hyperparathyreoidismus, Urin-pH-Tagesprofil und 2 × 24-h-Sammelurinuntersuchungen). Bei einigen Kalziumphosphatsteinbildnern besteht ein hohes Risiko für ein Rezidiv.

Kalziumphosphatsteine kommen hauptsächlich in zwei völlig unterschiedlichen Mineralien vor: Karbonatapatit und Brushit. Karbonatapatitkristalle fallen bei hohen Urin-pH-Werten > 6,8 aus und sind daher häufig infektassoziiert. Eine vollständige Steinsanierung ist dementsprechend unabdingbar und muss mittels regelmäßiger Urinuntersuchung nachkontrolliert werden. Brushitsteine entstehen hingegen in einem engen Urin-pH-Bereich von 6,5–6,8 und ihr Auftreten ist nicht mit einer Harnwegsinfektion verbunden. Die Brushitkristallbildung benötigt zudem eine hohe Konzentration an Kalzium und Phosphat im Urin.

### Harnsäuresteine (reine Harnsäure)

Die Bildung von Harnsäuresteinen wird begünstigt durch Hyperurikosurie und saures Milieu. Die metabolische Diagnostik der Harnsäuresteinbildung stützt sich auf ein Urin-pH-Tagesprofil, in dem sich typischerweise eine Säurestarre (Urin-pH-Werte konstant < 5,8) findet. Des Weiteren wird im 24-h-Sammelurin neben dem Volumen und der Harndichte die Harnsäureausscheidung quantifiziert. Die Bestimmung der Harnsäure im Serum erfolgt bereits mit den Basisuntersuchungen.

Die Bildung von Harnsäuresteinen wird begünstigt durch Hyperurikosurie und saures Milieu

Neben einer Steigerung der Trinkmenge zur Erzielung eines Harnvolumens von > 2,5–3 l/Tag sollten Harnsäuresteinbildner die Aufnahme von tierischem Eiweiß beschränken, um dadurch die Purinzufuhr mit der Nahrung zu reduzieren. Zur Korrektur des Urin-pH werden Alkalizitrate oder alternativ Natriumbikarbonat verwendet. Idealerweise sollte der pH-Wert im Urin auf Werte zwischen 6,2 und 6,8 eingestellt werden. Zur Chemolitholyse von Harnsäuresteinen wird eine stärkere Alkalisierung mit Urin-pH-Werten zwischen 7,0 und 7,2 angestrebt [4, 12]. Dabei gilt die Selbstkontrolle des Urin-pH durch den Patienten als wichtiges Mittel der Nachsorge, um zirkadiane Schwankungen zu erkennen und die Medikamente dementsprechend anzupassen. Bei nachgewiesener Hyperurikosurie sollte der Harnsäurespiegel durch Allopurinol 100 mg/Tag gesenkt werden. Im Falle einer begleitenden Hyperurikämie liegt die tägliche Dosis zwischen 100–300 mg [4]. Alternativ zu Allopurinol kann ebenso mit Febuxostat eine Normalisierung der Hyperurikämie und Hyperurikosurie erzielt werden [13].

### Struvitsteine

Struvitsteine bestehen chemisch aus Magnesiumammoniumphosphathexahydrat und bilden häufig im Gemisch mit Karbonatapatit Infektsteine, ausgelöst durch ureasebildende Keime im Urin. Da Harnwegsinfektionen ätiologisch ursächlich für die Infektsteinbildung sind, beschränkt sich in diesen Fällen die erweiterte metabolische Diagnostik und Nachsorge auf die Durchführung eines Urin-pH-Tagesprofils und einer Urinkultur. Die Anfertigung eines Antibiogramms ist essentiell, um eine gezielte antibiotische Therapie einzuleiten. Da bei einem Teil der Patienten das Keimspektrum der Urinkultur von den an den Konkrementen anhaftenden Keimen differiert, empfiehlt sich zusätzlich die mikrobiologische Untersuchung der entfernten Konkremente [14].

Wesentlicher Bestandteil der Rezidivprävention ist eine komplette Steinsanierung, da an zurückgelassenen Restfragmenten weiterhin Bakterien anhaften können, die dann zu einer Reinfektion und erneutem, oft raschem Steinwachstum führen können. Ebenso wichtig für die Prophylaxe ist die antibiotische Therapie der Harnwegsinfektion. Die Wahl des Antibiotikums basiert auf dem Antibiogramm der Urin- und/oder Steinkultur. Nach erfolgreicher Therapie der Infektion sollten die Patienten engmaschig nachkontrolliert werden, um bei erneutem Harnwegsinfekt rasch eine antibiotische Therapie einzuleiten.

### Zystinsteine

Die Zystinsteinbildung beruht auf der autosomal-rezessiv vererbten Zystinurie. Diagnostisch ist die Steinanalyse richtungsweisend, da Zystinsteine ausschließlich bei einer Zystinurie vorkommen. In der erweiterten metabolischen Diagnostik werden neben dem Harnvolumen und der Harndichte das Urin-pH-Tagesprofil und die Zystinausscheidung im 24-h-Sammelurin ermittelt [15]. Die engmaschige Nachkontrolle von Zystinsteinpatienten in einer spezifischen Sprechstunde ist zwingend, da die Rezidivhäufigkeit hoch ist und dementsprechend die metaphylaktischen Maßnahmen (Trinkmenge pro Tag von mindestens 3,5 l, Harnalkalisierung auf pH-Werte > 7,5 [6], medikamentöse Zystinsenkung durch α‑Mercaptopropionylglycin) überprüft werden müssen.

## Bildgebende Nachsorge

Nach erfolgter Therapie einer Urolithiasis (konservativ oder interventionell) ist eine Bildgebung indiziert, um den Therapieerfolg zu bestätigen sowie eine Obstruktion der ableitenden Harnwege auszuschließen. Dabei stellen sich zwei entscheidende Fragen: 1. Welche Bildgebung? und 2. Ist sie bei asymptomatischen Patienten wirklich notwendig?

Nach Therapie einer Urolithiasis ist eine Bildgebung indiziert, um den Therapieerfolg zu bestätigen

Prinzipiell gibt es mehrere geläufige Verfahren der Bildgebung zur Nachsorge nach Steintherapie: die Ultraschalluntersuchung, die konventionelle Röntgenleerbilduntersuchung (Rx Abdomen), die intravenöse Urographie (IVU) sowie die Computertomographie (CT). Dabei ist die CT ohne Verwendung von Kontrastmittel („non-contrast enhanced computed tomography“, NCCT) die sensitivste Methode, um Nieren- und Harnleitersteine zu detektieren und wird dementsprechend auch gemäß internationalen Richtlinien den anderen Verfahren vorgezogen [6, 7, 16]. Moderne niedrig dosierte CT („low dose NCCT“) konnten die Strahlenbelastung deutlich reduzieren, wobei eine hohe Sensitivität und Sensibilität von 96,6 % respektive 94,9 % beibehalten werden konnte [17]. Die Strahlendosen bei Anwendung moderner Low-dose-NCCT-Protokolle liegen zwischen 0,97 und 1,9 mSv, was ähnlichen Werten wie deren von Abdomenleeraufnahmen entsprechen, jedoch deutlich unterhalb der Strahlenbelastung der IVU liegen (1,3–3,5 mSv) liegen [18]. Neuere Verfahren mit deutlich reduzierter Strahlenbelastung jedoch besserer Steindetektion als die Abdomenleeraufnahme wie die Lodox-Bildgebung sind zwar vielversprechend, allerdings in klinischen Studien noch nicht genügend evaluiert, um im klinischen Alltag Einzug gehalten zu haben [19].

Im Zentrum der Debatte, ob eine Bildgebung bei asymptomatischen Patienten notwendig ist, steht die Frage, wie zuverlässig Beschwerdefreiheit eine Obstruktion des oberen Harntraktes ausschließt. Mehrere Studien haben gezeigt, dass beschwerdefreie Obstruktionen des oberen Harntraktes nach Steintherapie äußerst rar sind (0,4–4 %; [20, 21]). Somit muss eine beträchtliche Anzahl Nachkontrollen erfolgen, um eine einzige stumme Obstruktion zu entdecken und zu behandeln, weswegen die Modalität der Bildgebung der klinischen Situation angepasst werden sollte. Dies gilt v. a. im Hinblick auf eine Strahlenbelastung, die bei Steinbildnern aufgrund der wiederkehrenden Bildgebungen nicht zu unterschätzen ist.

Als absolute Mindestanforderung der bildgebenden Nachkontrolle gilt bei asymptomatischen Patienten jedoch eine Ultraschallbeurteilung des oberen Harntraktes nach erfolgter Steintherapie. Die Daten bezüglich des idealen Zeitpunkts der Kontrolle sind jedoch nicht eindeutig. Eine zu frühe Kontrolle kann dazu führen, dass Restfragmente, welche spontan und ohne Beschwerden abgehen könnten, zu einer Übertherapie, eine zu späte Kontrolle wiederum zu einem irreversiblen Verlust eines Teils der Nierenfunktion aufgrund einer obstruktiven Nephropathie führen. Gemäß verfügbarer Datenlage erscheint generell eine Kontrolle nach 4 bis 6 Wochen als ideal [22, 23], wobei die deutsche S2k-Leitlinie die Kontrolle innerhalb von 3 Monaten empfiehlt [4].

### Nach nicht-interventioneller Steintherapie

Patienten unter konservativer respektive medikamentös-expulsiver Steintherapie bedürfen einer Verlaufskontrolle, um den Erfolg der Therapie zu überprüfen. Steine < 5 mm gehen zu 75 % spontan ab, Steine ≥ 5 mm zu 62 %, wobei die Zeit bis zum Spontanabgang größenbedingt und sehr variabel ist (6–29 Tage, im Durchschnitt 17 Tage; [24]). Dementsprechend scheint eine Kontrolle nach ca. 4 Wochen angemessen, um eine allfällige Obstruktion zeitgerecht zu erkennen und zu behandeln. Bei symptomatische Patienten (engmaschige Nachkontrolle, evtl. auch mittels Low-dose-NCCT) und Patienten mit Spontansteinabgang und Steinasservierung bei Einzelstein (keine CT-Nachkontrolle notwendig) ist die klinische Situation relativ klar. Unklare Situationen stellen jedoch asymptomatische Patienten ohne gesicherten Spontansteinabgang dar. Bei dieser Patientengruppe sollte selbst bei unauffälligem Ultraschall des oberen Harntraktes ein Low-dose-NCCT evaluiert werden, um je nach Befund den Steinabgang zu bestätigen oder die weitere Steintherapie zu planen.

### Nach extrakorporaler Stoßwellenlithotripsie

In der Nachsorge nach extrakorporaler Stoßwellenlithotripsie (ESWL) sollte zwischen der kurz- und mittelfristigen postinterventionellen Bildgebung unterscheiden werden. In der S2k-Leitlinie zur Urolithiasis [4] besteht die Empfehlung zur kurzfristigen periinterventionellen radiologischen Kontrolle zur Überprüfung einer suffizienten Desintegration. Diese Bildgebung erfolgt meist am Folgetag der ESWL und erlaubt es, Patienten mit einer insuffizienten Desintegration kurzfristig einer erneuten ESWL zuzuführen und somit den Behandlungserfolg zu steigern. Diese Option bietet sich bei nicht-röntgendichten Steinen (Harnsäure‑, Xanthin‑, Ammoniumuratsteinen etc.) nicht an. Als Alternative kann ein Low-dose-NCCT durchgeführt werden, jedoch ist dies aufgrund der erhöhten Strahlenbelastung selten zu rechtfertigen. Ebenso sollte kurzfristig ein Ultraschall der Niere nach ESWL erfolgen, um allfällige Nierenhämatome oder Obstruktionen des oberen Harntraktes zu diagnostizieren.

Im weiteren Verlauf wird der definitive Behandlungserfolg evaluiert. Vor Zeiten des zunehmend verbreiteten Verfahrens der Low-dose-NCCT erfolgte die Erfolgskontrolle nach ESWL üblicherweise nach 6–12 Wochen mittels Abdomenleeraufnahme und Ultraschall des oberen Harntraktes. In der Literatur werden aufgrund von möglichem Abgang von Restfragmenten Verlaufskontrollen von bis zu 24 Monaten nach initialer Therapie beschrieben [25]. Angesicht modernerer Behandlungsmethoden mit schnellerem Behandlungserfolg ist dieses Vorgehen gegenüber Patienten immer schlechter zu vertreten, weswegen meistens anhand der Nachkontrolle nach 6–12 Wochen bereits entschieden wird, ob eine weitere Therapie durchgeführt werden soll.

### Nach Ureterorenoskopie

In der Nachsorge nach Ureterorenoskopie (URS) ist die Komplexität des initialen Eingriffs ausschlaggebend für die postoperative Bildgebung. In klinisch einfachen Situationen mit einem einzigen Konkrement, welches ohne Lithotripsie entfernt werden konnte, reicht ein Ultraschall des oberen Harntraktes für die Nachkontrolle aus (Ausschluss Obstruktion). Dieser erfolgt im Regelfall 6–12 Wochen nach der URS [26]. Bei symptomatischen Patienten oder persistierender Obstruktion ist eine weiterführende Bildgebung mittels CT (gegebenenfalls auch mit Kontrastmittel) indiziert, um eine allfällige Restfragmente respektive die Ursache der Obstruktion zu erkennen.

In klinisch komplexeren Situationen, welche eine Lithotripsie erfordern, eine erhöhte Steinlast besteht, anatomische Besonderheiten oder intraoperativ schlechte Sichtverhältnisse bestehen, kann eine zum Ultraschall zusätzliche radiologische Bildgebung wertvolle Informationen liefern, welche für die weitere Therapie wegweisend seien können. Obwohl bei röntgendichten Steinen eine Abdomenleeraufnahme häufig weitere Informationen liefert, wird doch aufgrund der deutlichen Strahlenreduktion meist ein Low-dose-NCCT vorgezogen.

### Nach perkutaner Nephrolithotomie

Die perkutane Nephrolithotomie (PNL) ist nach wie vor das Standardverfahren bei großen Nierensteinen, insbesondere (partiellen) Ausgusssteinen. Aufgrund des perkutanen Zugangs sowie der in der Regel rigiden Renoskope sind der Ureter sowie gewisse Kelchgruppen (je nach Punktionsort) nicht oder nur schlecht einsehbar. Somit besteht ein erhöhtes Risiko, dass Restfragmente übersehen werden. Durch die intraoperative Verwendung von Durchleuchtung (ohne und ggf. mit Kontrastmittel) sowie zusätzlich flexiblen Instrumenten (Zystoskop, Ureterorenoskop) können diese Mängel teilweise überwunden werden.

Wird im Rahmen der PNL eine Nephrostomie eingelegt, erfolgt als erste postoperative Bildgebung häufig eine antegrade Darstellung innerhalb von 2–10 Tagen. Somit können Restfragmente sowie der Abfluss des oberen Harntraktes beurteilt werden. Bei gutem Abfluss und Steinfreiheit kann die Nephrostomie praktisch risikofrei entfernt werden. Die weitere Nachsorge gestaltet sich analog der URS mittels frühzeitigem Ultraschall des oberen Harntraktes, wobei zusätzlich niederschwellig nach Restfragmenten gesucht werden soll. Zudem sollte aufgrund der bei perkutanen Steintherapien in der Regel komplexen Steinsituation eine zusätzliche Bildgebung mittels Abdomenleeraufnahme oder Low-dose-NCCT dringend erwogen werden.

### Nachsorge und Therapie von Residualfragmenten nach Steintherapie

Restfragmente im Ureter, welche 4–6 Wochen nach Therapie persistieren, bedürfen einer interventionellen Therapie, um den ungehinderten Urinabfluss aus dem oberen Harntrakt sicher zu stellen, da die Wahrscheinlichkeit für einen Spontanabgang unabhängig der Fragmentgröße reduziert ist. Restfragmente in der Niere führen selten zu Symptomen und können so unentdeckt bleiben, sofern sie nicht aktiv gesucht werden. Unabhängig der Steinkomposition führen Restfragmente in 21–59 % aller Patienten innerhalb von 5 Jahren zu einer erneuten Therapie. Bei Restfragmenten von > 5 mm ist ein Eingriff wahrscheinlicher als bei kleineren Fragmenten [27]. Das Rezidivrisiko bei Restfragmenten von Infektsteinen ist deutlich höher als bei anderen Steinkompositionen [28]. Bei postinterventionell absoluter Steinfreiheit wird von Rezidivraten von 0–10 % berichtet, bei persistierenden Restfragmenten von Rezidivraten von 40–85 % [29]. Es ist somit empfehlenswert, postinterventionell aktiv mittels Bildgebung nach Restfragmenten zu suchen und diese gegebenenfalls auch zu therapieren, um das Rezidivrisiko zu senken.

Regelmäßige Nachkontrollen sowie die Steinmetaphylaxe können zwar Steinrezidive nach Steintherapie nicht verhindern, jedoch kann die Rezidivhäufigkeit durch eine engmaschige Betreuung der Patienten deutlich gesenkt werden [2]. Dies hat nicht nur einen positiven Einfluss für den Patienten, sondern kann letztlich auch zur Kostensenkung im Gesundheitswesen beitragen. Gute Daten hierüber fehlen jedoch. Generell wird eine jährliche Nachkontrolle empfohlen. Die Frequenz der Nachkontrollen sollte aber zwingend anhand der Wahrscheinlichkeit für ein Steinrezidiv (Anzahl und Frequenz der Rezidive, Steinzusammensetzung, durchgeführte Metaphylaxe u. a.) angepasst werden, denn „one size fits all“ gilt weder bei der Steintherapie noch bei der Steinnachsorge und Metaphylaxe.

## Fazit für die Praxis

- Die Urolithiasis ist eine sehr häufige Erkrankung und deren Inzidenz steigt weltweit stetig an.
- Die westliche Lebensweise begünstigt das Auftreten der Urolithiasis; man kann von einer Zivilisationskrankheit sprechen.
- Die Urolithiasis geht häufig mit Grunderkrankungen einher, deren Früherkennung und Therapie wichtig sind (u. a. metabolisches Syndrom, Osteoporose).
- Die Metaphylaxe ist äußerst wichtig. Sie ist wie die metabolische und bildgebende Nachsorge individuell, d. h. Stein‑, Therapie- und Patientenfaktoren angepasst.
